# Vancomycin-resistant *Enterococcus faecium* pneumonia in a uremic patient on hemodialysis: a case report and review of the literature

**DOI:** 10.1186/s12879-020-4892-4

**Published:** 2020-02-22

**Authors:** Fengqin Li, Yonglan Wang, Linlin Sun, Xiaoxia Wang

**Affiliations:** Department of Nephrology, Shanghai Tongren Hospital, Shanghai Jiao Tong University School of Medicine, 1111 Xianxia Road, Shanghai, 200336 China

**Keywords:** Vancomycin resistant, *Enterococcus faecium*, Pneumonia, Epilepsy

## Abstract

**Background:**

Even though enterococci can cause serious infections in multiple sites, they are a rare cause of pneumonia. We reported a uremic patient with vancomycin-resistant *E. faecium* (VRE-fm) pneumonia, possibly related to epileptic seizures.

**Case presentation:**

A 57-year old man with uremia on hemodialysis was admitted to the hospital with complaint of recurrent epileptic seizures, followed by a two-week history of recurrent fever and cough with purulent sputum. Chest CT demonstrated multiple exudation of both lungs. He was diagnosed as community acquired pneumonia. Despite antibiotic combination therapy, abnormal chest shadows aggravated. Sputum and blood cultures were initially negative, but later blood culture grew VRE-fm. We suspected aspiration of gastrointestinal content induced by epilepsy as the most likely mechanism. The patient was successfully treated with a four-week course of linezolid according to the antibiotic susceptibility testing.

**Conclusions:**

Physicians should consider multi-drug resistant organisms such as VRE in uremic patients with pneumonia that fails to resolve with broad-spectrum antibiotics, especially in the cases with aspiration induced by epilepsy, immunocompromised conditions, and repeated or prolonged hospitalizations.

## Background

Enterococci are Gram-positive cocci which normally inhabit the intestinal tract of humans. *E. faecalis and E. faecium* are the most common strains. They started appearing as common pathogens in nosocomial infections in the 1970s. At the same time, antibiotic resistance among them started increasing [1]. Vancomycin-resistant *E. faecium* (VRE-fm) are multi-drug resistant micro-organisms, and the treatment options and infection control measures are limited. Additionally, there is a low clinical awareness. Therefore, infections caused by VRE-fm are a unique challenge to the clinician. Even though the most commonly reported Enterococcus infections are intra-abdominal infections, urinary tract infections, bacteremia and endocarditis, pneumonia is rarely described [2]. In this case report, we aimed to present a uremic patient with VRE-fm pneumonia, possibly related to epileptic seizures, being treated successfully with linezolid.

## Case presentation

A 57-year old man with uremia on hemodialysis complicated by severe renal anemia, hypertension and heart failure, presented with a two-week history of recurrent fever and cough with purulent sputum. Chest pain, nausea, vomiting, abdominal pain, diarrhea and night sweat was denied. He had been diagnosed with end-stage renal disease (ESRD) nearly 2 months ago, and the etiology was primary glomerulonephritis. Hemodialysis with the central venous catheter started at that time. Two weeks before admission, he suffered from recurrent epileptic seizures, characterized by convulsions of the whole body, unconsciousness and fecal incontinence in other hospital. Shortly afterwards, fever and cough with yellow phlegm gradually appeared. His labs were notable for white blood cell count (WBC) of 11.6 × 10 ^9^ /L with 87% neutrophils, hemoglobin (Hb) 61 g/L, and C-reactive protein (CRP) 74.06 mg/L. Blood and sputum cultures were negative. Computed tomography (CT) scan of the chest revealed pneumonia. Intravenous vancomycin (0.5 g three times a week) and meropenem (0.5 g Q8H) was administered empirically for suspected aspiration (given his lethargy after epileptic seizure). Then the symptoms improved gradually and indicators of infection dropped to normal during 1 week. Unexpectedly, the patient had fever again on the day of admission, with a temperature of 38.8 °C, accompanied by deteriorating general status. Therefore, he was transferred to our hospital for further treatment.

**Fig. 1:**
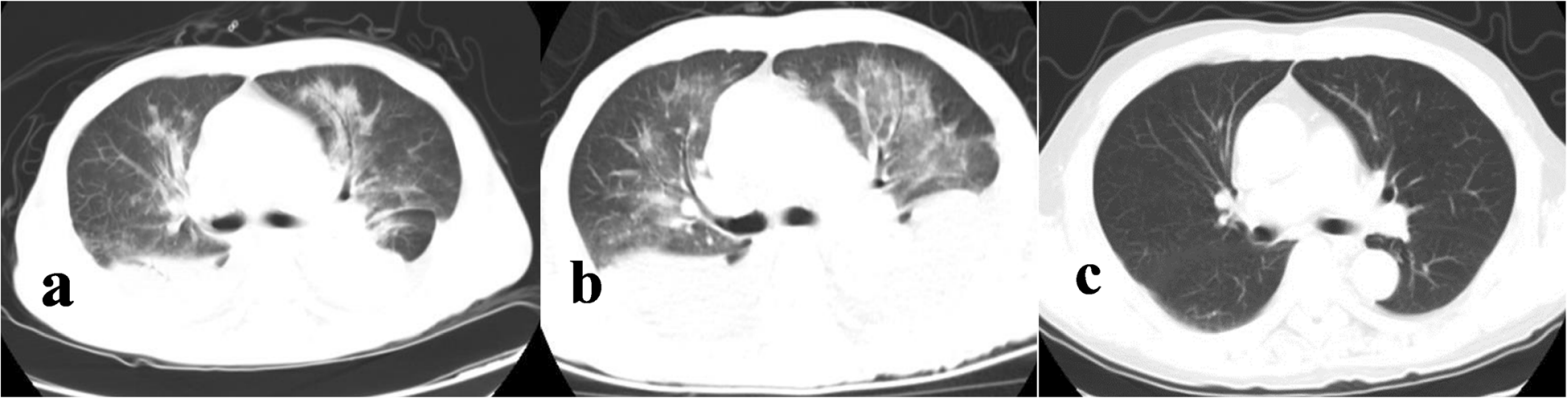
Characteristics of Chest CT during treatment. Chest CT showed infiltrates on both lungs on admission (**a**) and the infiltrates increased on day 7 after adjusting antibiotics treatment (**b**). Four weeks after starting linezolid therapy, the infiltrates disappeared (**c**)

**Fig. 2:**
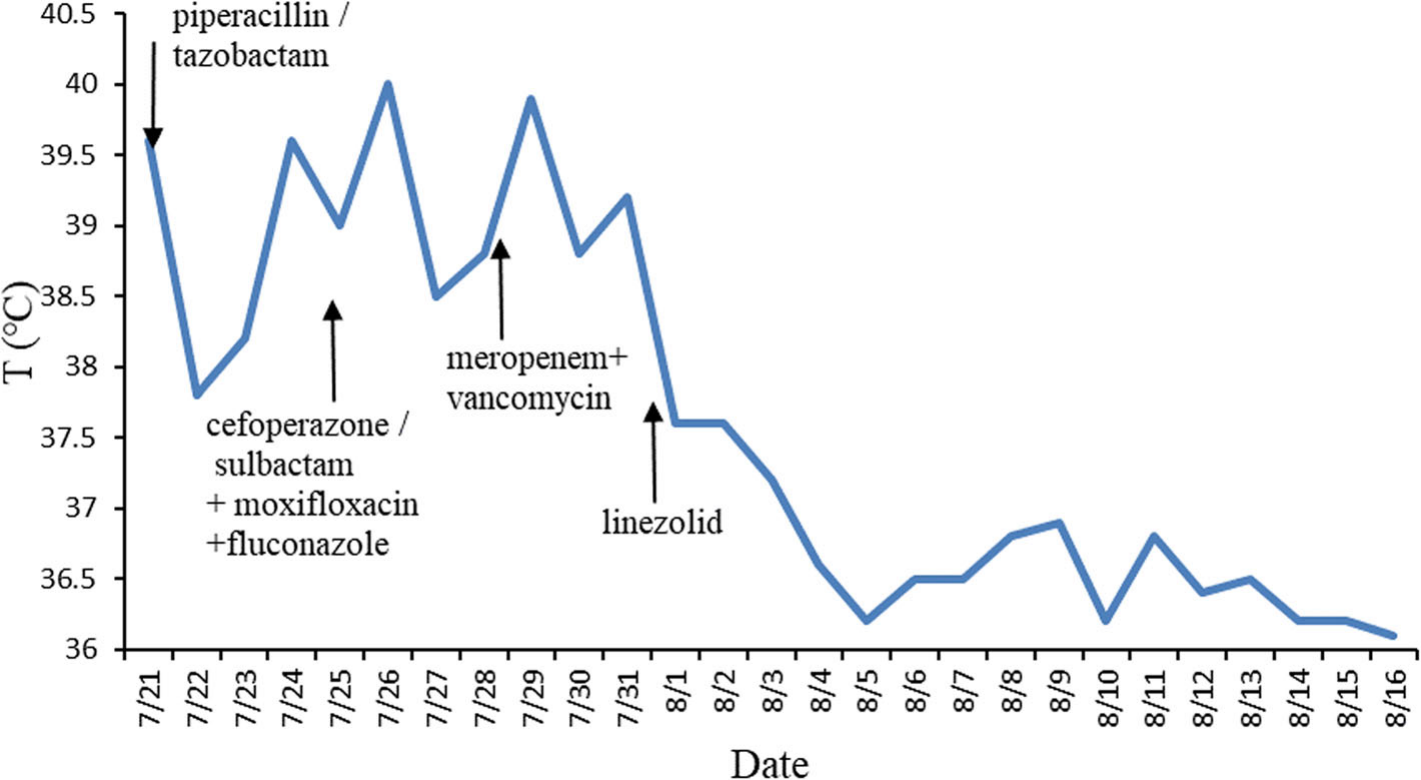
Response of the body temperature to antibiotic therapy. Despite of antibiotic therapy, piperacillin / tazobactam, cefoperazone / sulbactam+ moxifloxacin + fluconazole, and vancomycin + meropenem being used successively, the fever still persisted. Finally, the body temperature dropped to normal after linezolid therapy, according to the fourth blood culture and drug sensitivity result

**Fig. 3:**
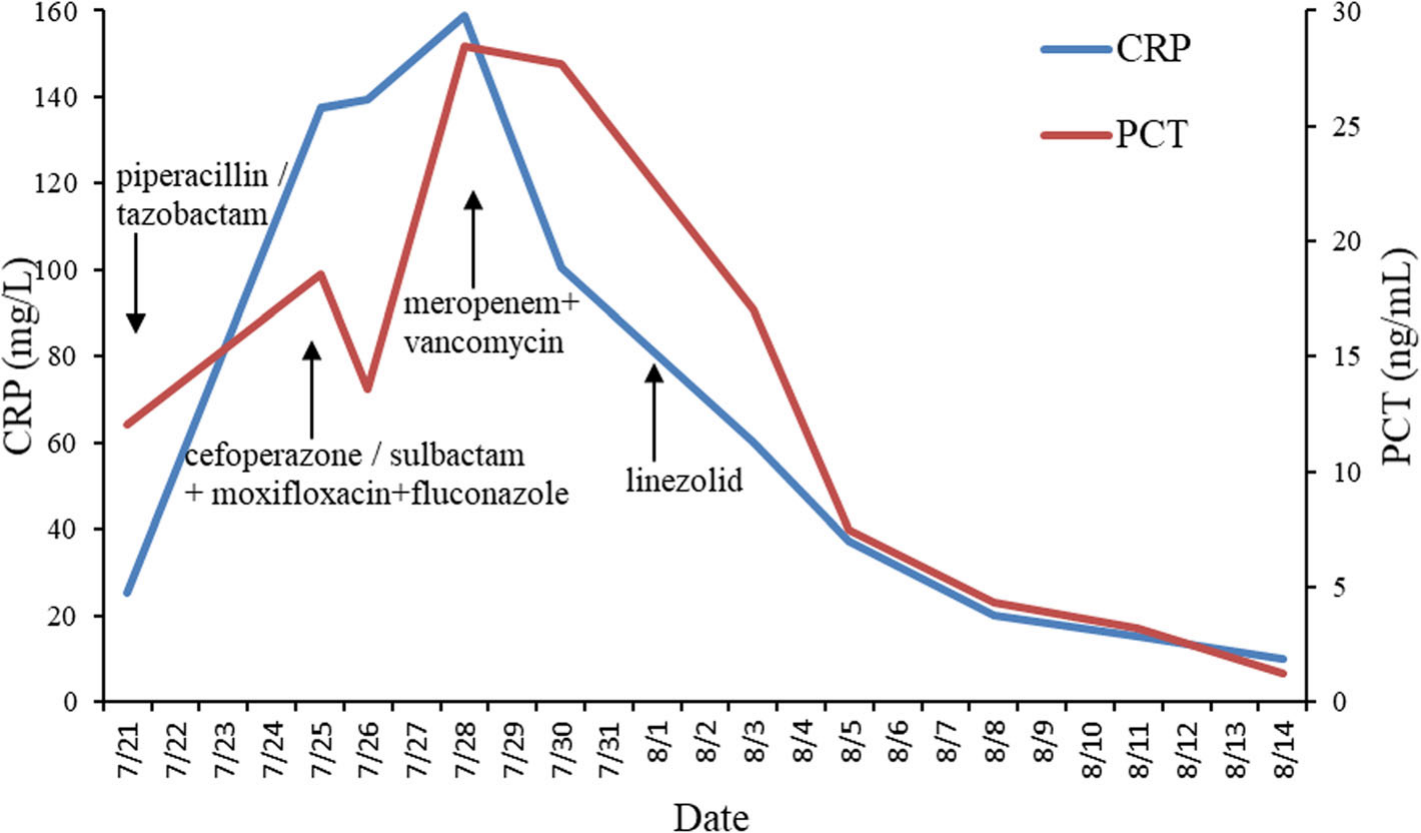
Response of infection indicators to antibiotic therapy. Despite of empirical antibiotic therapy, piperacillin / tazobactam, cefoperazone / sulbactam+ moxifloxacin + fluconazole, and vancomycin + meropenem being used successively, the levels of infectious indicators including CRP and PCT remained high. Finally, they decreased steadily after linezolid therapy, according to the fourth blood culture and drug sensitivity result

Physical examination was significant for appearance of severe anemia, a temperature of 38.8 °C, decreased breath sounds at the lung bases bilaterally, a diffuse moist rale on respiratory exam and a slight exudation around the right jugular hemodialysis catheter. Initial laboratory investigations revealed WBC of 7.0 × 10 ^9^ /L with 74.2% neutrophils, Hb 55 g/L, CRP 25.29 mg/L, procalcitonin (PCT) 3.02 ng/ml, Scr 557.1umol/L and B-type natriuretic peptide (BNP) greater than 5000 pg/ml. Chest CT demonstrated multiple exudation of both lungs, bilateral pleural effusion and atelectasis of both lower lobes (Fig. 1a). No valvular vegetation was found in echocardiography, and left ventricular ejection fraction (LVEF) was 43%. Initial diagnosis of admission was ESRD with hemodialysis, sever renal anemia, community acquired pneumonia, heart failure and suspected catheter-related infection. Treatment with intravenous piperacillin / tazobactam (2.25 g Q12H) for pneumonia and correction of heart failure and anemia were commenced. The dialysis catheter and urinary catheter were removed and peripheral blood cultures were collected. But his fever with a maximum temperature of 40 °C still persisted. Three consecutive blood, sputum culture and catheter cultures were negative. Additionally, thoracentesis and drainage were performed and hydrothorax culture was also negative. Laboratory detection of tuberculosis and fungi was all negative. Antibiotics were switched successively to cefoperazone sodium / sulbactam sodium (1.5 g Q12H) + moxifloxacin (0.4 g/day) + fluconazole (0.2 g/day), and vancomycin (0.5 g three times a week) + meropenem (0.5 g Q8H), according to consultation results of respiratory department. However, no apparent improvement was noted, and his general condition deteriorated progressively. Response of temperature and indicators of infection to antibiotic therapy was shown in Fig. 2 and Fig. 3. A repeated chest CT showed increased multiple exudation of both lungs (Fig. 1b). Finally, fourth blood culture became positive for *E. faecium* at>10^5^ CFU/ml (vanA genotype) on hospital day 13. At that point, antibiotic therapy was switched to intravenous linezolid (600 mg Q12H) based on the sensitivity pattern of isolates that were vancomycin, moxifloxacin, gentamicin, penicillin, rythromycin and ampicillin resistant, and linezolid, teicoplaninand and tegafycline sensitive. In the following days, the fever subsided gradually (Fig. 2). And the CRP and PCT levels decreased steadily (Fig. 3). The patient completed a four-week course of linezolid with complete resolution of chest CT abnormalities (Fig. 1c).

## Discussion and conclusion

*E. faecalis* is more pathogenic than *E. faecium*, but the latter exhibits more resistance, taking up the majority of VRE infections [3]. VRE is an important nosocomial pathogen spreading in hospitals worldwide. It was reported by the National Healthcare Safety Network (NHSN) that 35.5% of enterococcal hospital-associated infections were resistant to vancomycin from 2009 to 2010, which ranked as the second most common cause of nosocomial infections in the US [3]. Additionally, Markwart et al. reported the proportion of VRE-fm was increasing from 11.2% in 2014 to 26.1% in 2017 in German hospitals, particularly in southern regions in Germany [4]. In china, there was a rapid increase in vancomycin resistance from 12.4% in 2007 to 42.0% in 2016 among nosocomial enterococcal isolates in ICU, according to the Taiwan Nosocomial Infection Surveillance System [5]. However, pulmonary infections due to enterococcus are distinctly unusual. A prospective and observational study of 110 patients with serious infections due to Enterococcus across six hospitals found that there was 4% of those infections located in the respiratory tract over the course of 1 year [6]. The best evidence that enterococcal pneumonia is rare has been reported by Richards et al. They found only two cases of enterococcal pneumonia among 181,993 patients evaluated for a total of 715,930 patient-days in American medical intensive care units [7]. Recently, according to the National Healthcare Safety Network, only 1% of ventilator-associated pneumonias were caused by Enterococcus spp. [8].

We conducted a literature search based on PubMed in an attempt to identify all published cases of pleuropulmonary infection due to enterococcus, without time limits. Twenty-four cases of Enterococcus-associated pleuropulmonary infection previously published in the literature were eventually summarized in Table 1 [6, 9–26]. The enterococcal pneumonia cases previously described in the literature mainly occurred in elderly patients with immunosuppression, organ transplantation, hematological malignancies, solid cancer, renal failure, cardio-cerebrovascular disease and chronic obstructive pulmonary disease. Ten of the 24 cases of Enterococcus pneumonia were complicated by lung abscess and empyema requiring drainage. There were 8 cases of pneumonia due to *E. faecium* of which 3 were vancomycin-resistant. To our knowledge, ours is the first VRE-fm pneumonia after epileptic seizure in a patient with ESRD on hemodialysis. Interestingly, the patient had recurrent epilepsy before he developed pneumonia, which raised the likelihood that aspiration of gastrointestinal content induced by epilepsy (given his altered mental status and lethargy) occurred at some point. And his pulmonary infiltrates involved the middle and lower parts of two lungs, which were typical for aspiration mechanism. According to previous reports, Enterococcal-associated aspiration pneumonia also occurred in patients fed by Dobb-Hofftube and experiencing near drowning [23, 26]. Since our patient was admitted with a hemodialysis catheter accompanied by purulent exudation at the outlet of the catheter, recurrent fever, heart failure and positive blood culture, another possible mechanism that pneumonia secondary to a septic embolus originated from the dialysis catheter or the heart was suggested. However, imaging of vessels at the catheter and an echocardiography showed no embolism and the catheter culture was negative in the current case. From the cases summarized in Table 1, it is notable that only 2 cases of Enterococcal pneumonia were associated with endocarditis [11, 13]. Furthermore, previous literature data reported 3 cases of VRE-fm pneumonia were treated with linezolid, of which 2 survived and 1 died. In contrast, the isolate recovered from our patient was sensitive to linezolid according to the drug sensitivity test, and his infection indicators and body temperature showed good response to the treatment of linezolid, and the chest CT abnormalities completely resolved after a four-week course of linezolid.

**Table 1:** Summary of identified cases of Enterococcus pleuro-pulmonary infections

Age (y) /Sex [Ref]	Co-morbidities	Community versus nosocomial	Pleuro-pulmonary infection	Positive cultures: Specimen/isolates	Resistance to	Antibiotic therapy	Outcome
39/female [9]	None	Community	Pneumonia	Sputum/Enterococcus + *Escherichia coli*	Streptomy-cin	Aeromycin (Chlortetra-cycline) + Streptomy-cin	Alive
54/male [10]	None	Community	lung abscess	Sputum/Enterococcus	None	Penicillin+Streptomycin	Alive
46/female [10]	PVD and CVD	Community	lung abscess	TBA/Enterococcus	Penicillin	Ampicillin+Gentamicin	Alive
NA [11]	NA	NA	Pneumonia	Sputum/Enterococcus	NA	NA	NA
85/male [12]	CVD	Nosocomial	Pneumonia	TTA/Enterococcus	Penicillin	Ampicillin	Died
54/male [12]	Hyponatremia	Nosocomial	Pneumonia	TTA/Enterococcus	Penicillin	Ampicillin	Alive
52/male [13]	NA	Nosocomial	Empyema	Pleural fluid/ Enterococcus	NA	NA	Alive
NA [14]	NA	Nosocomial	Pneumonia	Lung tissue / Enterococcus	NA	NA	NA
68/male [15]	None	Community	Pneumonia	PTA/*Enterococcus faecium*	Penicillin Ampicillin Tobramycin	Ciprofloxacin	Alive
NA [5] (4 patients)	NA	Both	PneumoniaEmpyema	Sputum, BAL, PSB and pleura fluid / Enterococcus	NA	NA	NA
78/NA [16]	A.Fib, CVA and CAD	Nosocomial	PneumoniaEmpyema	NA/ Enterococcus	NA	amoxicillin/clavulanate	Alive
49/male [17]	Alcoholic hepatitis	Community	Pneumonia	PTA/*Enterococcus faecalis*	Penicillin, Ampicillin	Vancomycin	Alive
NA/NA [18]	Chronic hemodialysis	Community	Pneumonia	NA/Enterococcus faecium	Multidrug resistant	Vancomycin	Alive
68/female [8]	Lymphoma	Nosocomial	PneumoniaEmpyema	BAL, pleural fluid and blood/ Enterococcus faecium	ampicillin	Vancomycin+ Gentamicin	Died
29/male [19]	kidney transplant recipient	Nosocomial	Lung abscesses	Blood and BAL/ Enterococcus faecium	NA	teicoplanin linezolide	Alive
63/female [20]	CVA	Nosocomial	PneumoniaEmpyema	TTA and sputum/ Enterococcus faecalis	NA	Amoxicillin/clavulanate	Alive
81/male [21]	Renal carcinoma chronic renal failure	Nosocomial	Pneumonia	Blood and sputum/ Enterococcus faecalis	gentamicin	ampicillin	Alive
41/male [22]	HIV	Community	Pneumoniaempyema	Lung tissue/ Enterococcus faecium	Vancomyci-n	linezolid	Alive
[23] 73/male	pancreatic cancer diabetes	Community	Pneumonia	Sputum/ Enterococcus faecium	Ampicillin Levofloxacin	Vancomycin	Alive
75/male [24]	Asthma, chronic lymphocytic leukaemia	Nosocomial	Pneumonia	BAL and blood/ Enterococcus faecium	Vancomyci-n	linezolid and daptomycin	Died
67/male [25]	Asplenia, COPD, CVA and alcohol abuse	Community	Pneumoniaempyema	pleural fluid/ Enterococcus faecium	Ampicillin Vancomyci-n	linezolid	Alive

*NA* Not available, *CVD* Cerebrovascular disease, *CAD* Coronary artery disease, *PVD* Peripheral vascular disease, *BAL* Bronchoalveolar lavage, *PSB* Protected brush specimen, *TTA* Transtracheal aspiration, *PTA* Percutaneous transthoracic aspiration, *HIV* Human immunodeficiency virus, *A. fib* Atrial fibrillation; *CVA* Cerebrovascular accident, *COPD* Chronic obstructive pulmonary disease

Previous case reports of enterococcal pneumonia were based on clinical findings and infiltrate on chest X ray or CT in combination with the isolation of enterococci in cultures from a transtracheal aspirate, protected brush (PS), bronchoalveolar lavage (BAL), sputum or lung tissue. Since isolation of Enterococcus from respiratory secretions usually represents colonization, lung tissue, PS or BAL culture may identify true infection of the lower respiratory tract more accurately than the sputum or endotracheal aspirate cultures. However, in our case, bronchoscopy and lung needle biopsy was not performed to obtain lung tissue, PS or BAL specimens, because the patient was seriously ill at that time. Evidence supporting a diagnosis of VRE-fm pneumonia in our patient included persistent respiratory symptoms, multiple infiltration on chest CT, bacteremia with VRE-fm and good response to linezolid based on susceptibility testing.

Currently, nine phenotypes of vancomycin resistance described are van A, van B, van C, van D, van E, van G, van L, van M and van N [27]. Van A contributes to most of the human cases of VRE around the world, and is mostly carried by *E. faecium*. Moreover, a study by Bocanegra-Ibarias et al., which involved phenotypic and genotypic characterization of VRE-fm clinical isolates from two hospitals in Mexico, first detected VanB phenotype-vanA genotype [28]. Hypermutability, increased mobile genetic elements, metabolic alterations and hypermutability confer drug resistance to *E. faecium*. Our patient was found to be infected by *E. faecium* with Van A gene, so he was clinically unresponsive to vancomycin. The main causes for emergence of VRE-fm in our patient may be his immunocompromised conditions due to ESRD, repeated hospitalizations, mechanical instrumentation (invasive hemodialysis catheter) and exposure to multiple antibiotics (specifically vancomycin). Therefore, clinicians should minimize unnecessary invasive procedures and vancomycin abuse. De-escalation from initial broad-spectrum antibiotics to narrow spectrum antibiotics immediately after receiving the antibiotic sensitivity report is necessary. Additionally, strict adherence to infection control practices can prevent further emergence and spread of drug resistance.

In conclusion, VRE pneumonia is rarely reported. Physicians should consider multi-drug resistant organisms such as VRE in uremic patients with pneumonia that fails to resolve with broad-spectrum antibiotics, especially in the cases with aspiration induced by epilepsy, immunocompromised conditions, and repeated or prolonged hospitalizations. Better clinical outcomes can be expected if the empirical antibiotic treatment covers VRE and early adjustment of sensitive antibiotics based on susceptibility testing.

## Data Availability

The datasets used and/or analyzed during the case report are available from the corresponding author on reasonable request.

## Abbreviations

BAL: Bronchoalveolar lavage
BNP: B-type natriuretic peptide
CRP: C-reactive protein
CT: Computed tomography
ESRD: End-stage renal disease
Hb: Hemoglobin
pct: Procalcitonin
PS: Protected brush
VRE-fm: Vancomycin-resistant *E. faecium*
WBC: White blood cell count

## References

[1] Uttley AH, Woodford N, Johnson AP, Cookson B, George RC (1993). Vancomycin-resistant enterococci. Lancet.

[2] O'Driscoll T, Crank CW (2015). Vancomycin-resistant enterococcal infections: epidemiology, clinical manifestations, and optimal management. Infect Drug Resist.

[3] Sievert DM, Ricks P, Edwards JR, Schneider A, Patel J, Srinivasan A, Kallen A, Limbago B, Fridkin S (2013). Antimicrobial-resistant pathogens associated with healthcare-associated infections summary of data reported to the National Healthcare Safety Network at the Centers for Disease Control and Prevention, 2009–2010. Infect Control Hosp Epidemiol.

[4] Markwart R, Willrich N, Haller S, Noll I, Koppe U, Werner G, Eckmanns T, Reuss A (2019). The rise in vancomycin-resistant Enterococcus faecium in Germany: data from the German antimicrobial resistance surveillance (ARS). Antimicrob Resist Infect Control.

[5] Chen C, Lin L, Chang Y, Chang C (2017). Clinical and microbiological characteristics of vancomycin-resistant Enterococcus faecium bloodstream infection in Central Taiwan. Medicine (Baltimore).

[6] Patterson JE, Sweeney AH, Simms M, Carley N, Mangi R, Sabetta J, Lyons RW (1995). An analysis of 110 serious enterococcal infections. Epidemiology, antibiotic susceptibility, and outcome. Medicine (Baltimore).

[7] Richards MJ, Edwards JR, Culver DH, Gaynes RP. Nosocomial infections in medical intensive care units in the United States. Crit Care Med. 1999;27(5):887–92. 10.1097/00003246-199905000-00020 10362409

[8] Hidron AI, Edwards JR, Patel J, Horan TC, Sievert DM, Pollock DA, Fridkin SK (2008). Antimicrobial-resistant pathogens associated with healthcare-associated infections: annual summary of data reported to the National Healthcare Safety Network at the Centers for Disease Control and Prevention, 2006–2007. Infect Control & Hosp Epidemiol.

[9] Delank HW (1953). The disease picture in enterococcal pneumonia. Medizinische.

[10] Morris JF, Okies JE (1974). Enterococcal lung abscess: medical and surgical therapy. Chest.

[11] Tornos MP, Mayor G, Nadal A, Soler-Soler J (1984). Empyema and splenic abscess in infective endocarditis. Int J Cardiol.

[12] Maki DG, Agger WA (1988). Enterococcal bacteremia: clinical features, the risk of endocarditis, and management. Medicine (Baltimore).

[13] MacEachern P, Giannoccaro JP, Elsayed S, Read RR, Laupland KB (2005). A rare case of pleuropulmonary infection and septic shock associated with Enterococcus faecium endocarditis. J Inf Secur.

[14] Feeley TW, Du Moulin GC, Hedley-Whyte J, Bushnell LS, Gilbert JP, Feingold DS (1975). Aerosol polymyxin and pneumonia in seriously ill patients. N Engl J Med.

[15] Portero JL, Porcel JM, Ruiz A, Rubio-Caballero M (1994). Community-acquired pneumonia caused by Enterococcus faecium. Med Clin (Barc).

[16] Escudero SV, Adrados BG, Camara GT, Romerales RA (1995). Enterococcal empyema. An Med Interna.

[17] Molinos L, Gullon JA, Riesgo C, Dominguez MJ, Martinez J (1995). Community acquired pneumonia due to Enterococcus. An entity for consideration?. Enferm Infecc Microbiol Clin.

[18] Morii K, Takechi T, Shimizu Y (2002). A critical pneumonia by multidrug-resistant Enterococcus faecium in a chronic hemodialysis patient. A case report. Kansenshogaku Zasshi.

[19] Levora J, Teplan V, Viklicky O (2007). Enterococcus faecium as a cause of pulmonary abscesses in kidney transplant recipient. Transpl Int.

[20] Bergman R, Tjan DH, Schouten MA, Haas LE, van Zanten AR (2009). Pleural Enterococcus faecalis empyema: an unusual case. Infection.

[21] Grupper M, Kravtsov A, Potasman I (2009). Enterococcal-associated lower respiratory tract infections: a case report and literature review. Infection.

[22] Vanschooneveld T, Mindru C, Madariaga MG, Kalil AC, Florescu DF (2009). Enterococcus pneumonia complicated with empyema and lung abscess in an HIV-positive patient. Case report and review of the literature. Int J STD AIDS.

[23] Kimura Y, Kobayashi I (2011). A case of pneumonia due to Enterococcus faecium after near drowing. Kansenshogaku Zasshi.

[24] Abu OM, Abu GM, Kim S, Howell G: Strongyloides hyperinfection syndrome and VRE pneumonia. BMJ Case Rep. 2017. 10.1136/bcr-2016-216634. 10.1136/bcr-2016-216634 PMC5256491 28093424

[25] Cotton MJ, Packer CD (2018). Vancomycin-resistant Enterococcus faecium empyema in an Asplenic patient. Cureus.

[26] Berk SL, Verghese A, Holtsclaw SA, Smith JK (1983). Enterococcal pneumonia. Occurrence in patients receiving broad-spectrum antibiotic regimens and enteral feeding. Am J Med.

[27] Chen C, Sun J, Guo Y, Lin D, Guo Q, Hu F, Zhu D, Xu X, Wang M (2015). High prevalence of vanM in Vancomycin-resistant Enterococcus faecium isolates from Shanghai. China Antimicrob Agents Chemother.

[28] Bocanegra-Ibarias P, Flores-Trevino S, Camacho-Ortiz A, Morfin-Otero R, Villarreal-Trevino L, Llaca-Diaz J, Martinez-Landeros EA, Rodriguez-Noriega E, Calzada-Guereca A, Maldonado-Garza HJ (2016). Phenotypic and genotypic characterization of vancomycin-resistant Enterococcus faecium clinical isolates from two hospitals in Mexico: first detection of VanB phenotype-vanA genotype. Enferm Infecc Microbiol Clin.

